# Association studies of several cholesterol-related genes (ABCA1, CETP and LIPC) with serum lipids and risk of Alzheimer’s disease

**DOI:** 10.1186/1476-511X-11-163

**Published:** 2012-11-26

**Authors:** Zhijie Xiao, Juan Wang, Weirong Chen, Peng Wang, Houlin Zeng, Weixi Chen

**Affiliations:** 1 Department of Neurology, The Second XiangYa Hospital of Central South University, No.139 of People’s Middle Road, Changsha, Hunan Province 410011, People’s Republic of China; 2 Medical Center of Women and Children Health Hospital, Changsha, Hunan Province, 410011, People’s Republic of China

**Keywords:** Alzheimer’s disease, Lipids, ATP-binding cassette transporter A1, LIPC, Cholesteryl ester transfer protein, Single-nucleotide polymorphisms

## Abstract

**Objectives:**

Accumulating evidence suggested that dysregulation of cholesterol homeostasis might be a major etiologic factor in initiating and promoting neurodegeneration in Alzheimer’s disease (AD). ATP-binding cassette transporter A1 (ABCA1), hepatic lipase (HL, coding genes named LIPC) and cholesteryl ester transfer protein (CETP) are important components of high-density lipoprotein (HDL) metabolism and reverse cholesterol transport (RCT) implicated in atherosclerosis and neurodegenerative diseases. In the present study, we will investigate the possible association of several common polymorphisms (ABCA1R219K, CETPTaqIB and LIPC-250 G/A) with susceptibility to AD and plasma lipid levels.

**Methods:**

Case–control study of 208 Han Chinese (104 AD patients and 104 non-demented controls) from Changsha area in Hunan Province was performed using the PCR-RFLP analysis. Cognitive decline was assessed using Mini Mental State Examination (MMSE) as a standardized method. Additionally, fasting lipid profile and the cognitive testing scores including Wechsler Memory Scale (WMS) and Wisconsin Card Sorting Test (WCST) were recorded.

**Results and conclusions:**

We found significant differences among the genotype distributions of these three genes in AD patients when compared with controls. But after adjusting other factors, multivariate logistic regression analysis showed only ABCA1R219K (B = −0.903, *P* = 0.005, OR = 0.405, 95%CI:0.217-0.758) and LIPC-250 G/A variants(B = −0.905, *P* = 0.018, OR = 0.405, 95%CI:0.191-0.858) were associated with decreased AD risk. There were significantly higher levels of high-density lipoprotein cholesterol (HDL-C) and apolipoproteinA-I in the carriers of KK genotype and K allele (*P* < 0.05), and B2B2 genotype of CETP Taq1B showed significant association with higher HDL-C levels than other genotypes (*F* = 5.598, *P* = 0.004), while -250 G/A polymorphisms had no significant effect on HDL-C. In total population, subjects carrying ABCA1219K allele or LIPC-250A allele obtained higher MMSE or WMS scores than non-carriers, however, no significant association was observed in AD group or controls. Therefore, this preliminary study showed that the gene variants of ABCA1R219K and LIPC-250 G/A might influence AD susceptibility in South Chinese Han population, but the polymorphism of CETPTaq1B didn't show any association in despite of being a significant determinant of HDL-C.

## Introduction

Alzheimer' disease(AD) is one of the most common neurodegenerative diseases which hallmark is the deposition of amyloid-β (Aβ) in brain parenchyma and cerebral blood vessels, accompanied by progressive cognitive decline. However, detailed pathogentic mechanisms of AD remain a matter of speculation. With the exception of age and APOEε4 allele which is only one undisputed genetic component identified [1,2], environmental risk profiles including smoking, alcohol, obesity, hypertension, dislipidemia, diabetes and cardiovascular diseases may be candidates for AD [3-5]. Previous research has demonstrated that cerebral atherosclerosis was strongly associated with an increased frequency of neuritic plaques [6]. Moreover, the association of some gene variation in lipid metabolism with AD has been identified in a number of study [7,8]. So the close links between vascular pathology and AD have been payed more attention.

Accumulated evidence has shown that dysfunction of cholesterol metabolism may contribute to cognitive decline and AD [9], such as decreased high-density lipoprotein cholesterol (HDL-C) levels [10,11], increased low-density lipoprotein cholesterol (LDL-C) levels [4,12], and decreased apolipoproteinA-I (apoA-I) levels [11,13] known to be important risk factors for coronary atherosclerosis disease (CAD). The recent identification has been extensively recognized that the effect of serum total cholesterol (TC) on dementia risk occurs in midlife but not late-life, which may be different cardiovascular risk factor profiles for AD [14,15]. In addition, intervention of cholesterol-lowering drugs in some observational or cohort studies may decrease risk of developing dementia [16] and delay the onset of AD in cognitively healthy elderly individuals [17]. Growing evidence in previous animal and cellular studies has also suggested that abnormalities in cholesterol metabolism are important in the pathogenesis of AD, potentially by increasing neuronal content of cholesterol, cleavage of the amyloid protein precursor (APP), accumulation of Aβ peptide, neuroinflammation, impairment in the cholinergic system and working memory [18-20]. However, the mechanisms are still ill-defined. Recent work from Ghribi O and Marwarha G et al. indicated that the oxidized cholesterol metabolite, 27-hydroxycholesterol, which had the ability to cross into the brain, could increase Aβ levels and phosphorylated tau-protein in adult rabbit brain slices [21,22], and the same result also observed in cultured human neuroblastoma SH-SY5Y cells [23]. Sharma S et al. found that hypercholesterolemia-induced Aβ production in rabbit brain was associated with alteration in IGF-1 signaling [24].

Many regulating genes affecting cholesterol or lipoprotein function have been implicated in the pathogenesis of atherosclerosis diseases, especially reverse cholesterol transport (RCT) mediated by HDL-C. HDL biogenesis occurs through intergrated pathway that involves the lipid transporter ABCA1 interacting with lipid-free apoA-I in the initial steps, cholesteryl-ester transfer protein (CETP) transferring cholesteryl-esters to VLDL/LDL by the LDL receptor for eventual catabolism, lipoprotein lipase, hepatic lipase, and endothelial lipase contributing to the hydrolysis of phospholipids and residual triglycerides, and phospholipid transfer protein transferring phospholipids from VLDL/LDL to HDL, etc. [25]. ABCA1 is a trans-membrane protein responsible for the efflux of cholesterol and phospholipids to apolipoproteins such as apoA-I and apoE [26], the gene mutations of which has been recognized contributing to Tangier's disease and familial hypoalphalipoproteinemia [27]. As known as common polymorphisms in this gene could affect plasma HDL-C levels and the risk of atherosclerosis and CAD [28-30], dysregulation of ABCA1 expression may be a central mediator of AD neuropathology and dementia severity [31]. Several studies with AD mouse model revealed that ABCA1 expression could regulate the level of apoE lipidation and the production of Aβ [32-35]. But controversies still remained [36,37]. One of the most studied ABCA1 variants is R219K (rs2230806) in exon region. Previous researches indicated the common R219K variant was associated with CSF cholesterol levels [38].

Hepatic lipase (HL) is a lipolytic enzyme, synthesized by hepatocytes, not only participating in hydrolysis of triglycerides and phospholipids of plasma HDL and LDL, resulting in the formation of atherogenic small dense LDL and HDL_3_-C particles [39], but also playing an important role in RCT by its ability in the cellular uptake of remnant lipoproteins as a ligand in the liver [40]. CETP is a plasma glycoprotein that facilitates the transferring of cholesterol esters, triglycerides and phospholipids between plasma lipoproteins [41]. Common genetic variants at both the CETP and LIPC loci have been identified that may cause significant alterations in HDL-C level and atherosclerosis, however, the findings are still controversial in diverse racial groups [42-45]. The HL gene is located on chromosome 15 (q15–q22) in humans [46]. Four common LIPC promoter variants (−250 G/A, -514C/T, -710 T/C and -763A/G) which are in almost complete linkage disequilibrium have been widely studied [47]. The rare -250A and −514 T alleles have been associated with low HL activity [48,49], buoyant LDL particles [49], high triglyceride levels [50], high HDL-C levels, and decreased risk of CAD [50-52]. One of the most identified CETP variant is the TaqIB (rs708272) in the first CETP intron [53]. The minor “B2” allele (absence of the TaqIB restriction site) has been associated with increased HDL-C levels and decreased CETP activity [44].

As mentioned, abnormal lipid metabolism was consistently indicated in the pathogenesis of AD, thereby, such genetic variants may provide important functional candidates for AD. In the past decade, the relationship between ABCA1R219K variants and risk of AD have been reported in different regions and ethnic groups, however, conflicting results were noted [38,54-58]. Polymorphisms in the CETP gene have been associated with lower cardiovascular risk, but associations with memory decline and dementia risk are unclear. Chen DW et al. in Xuanwu Hospital of our country showed that there was no significant difference of CETPTaq1B polymorphism between AD and control groups regardless of APOE ε4 carrier status [59]. It also has been reported about the association between several LIPC SNPs with the risk of AD in two research [60,61], however, no significant difference was identified. Previously inconsistent results have appeared in the literature, hence, the epistatic effect of such variants on the risk profile associated with AD remains to be elucidated.

Accordingly, in this study we will determine the relationship of polymorphisms in several common HDL-C-related gene loci (ABCA1R219K, LIPC-250 G/A and CETP Taq1B) with plasma lipids and the risk of AD in Southern Chinese Han population.

## Material and methods

### Subjects

A total of 104 clinically diagnosed late-onset AD patients and 104 controls without cognitive impairment were randomly collected from a population of Chinese Han nationality in Changsha area. Mini-mental state examination (MMSE) was used as the measure of cognitive detecting and dementia severity [62]. All cases were sporadic AD patients without relationships in Chinese Han population of Hunan Province. Nobody had a family history of dementia. All patients were clinically evaluated and met the NINCDS/ADRDA criteria for diagnosed probable AD [63]. There were 57 men and 47 women in AD subjects, with an average age of 77.8±6.74 years and an average age-at-onset of 72.9±7.29 years. During the same period, all controls were recruited from old people above 65 years in the same region, without cognitive impairment and any severe nervous system diseases and mental illnesses. There were 56 men and 48 women. The mean age was 76.5±6.14 years. Basic physical examination, Wechsler Memory Scale Test and Wisconsin Cart Sorting Test were carried out for the whole subjects. Everyone was interviewed for a detailed inquiry including medical history, including family history, education, smoke, alcohol, hyperlipidemia, hypertension, diabetes and coronary heart disease, etc. A venous fasting blood sample of 5 ml was obtained from all subjects. The present study was carried out in compliance with the Helsinki Declaration and was approved by the Ethics Committee of the Second XiangYa Hospital of Central South University. Written informed consent was obtained from the patient for publication of this report and any accompanying images.

### Biochemical analysis

3 ml blood sample was used to determine serum lipid levels. The levels of TC, TG, HDL-C, and LDL-C in samples were determined by enzymatic methods, and serum apoA-I and apoB levels by the immunoturbidimetric immunoassay. All determinations were performed with an autoanalyzer in the Clinical Laboratory Center of the Second XiangYa Hospital of Central South University. According to NCEP-Adult Treatment Panel III [64], lipid parameters of all subjects were stratified presenting with appropriate HDL-C level (≥1.03 mmol/L[40 mg/dL]) or low HDL-C (<1.03 mmol/L); appropriate TC level (≤5.17 mmol/L[200 mg/dL]) or higher level(>5.17 mmol/L); appropriate TG level (≤1.70 mmol/L[150 mg/dL]) or higher level (>1.70 mmol/L); appropriate LDL-C level (≤2.59 mmol/L[100 mg/dL]) or higher level (>2.59 mmol/L).

### Genotying

**Figure 1:**
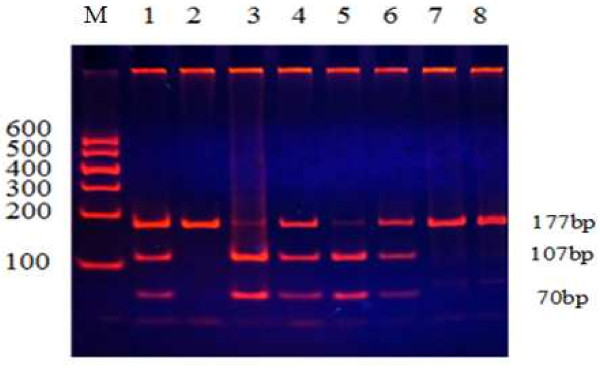
**Genotyping of PCR products of ABCA1R219K.** Lane M, 100 bp Marker Ladder; Lanes 1/4/6, RK genotype (177 bp, 107 bp and 70 bp); Lane 2/7/8, RR genotype (177 bp); and Lanes 3/5, KK genotype (107 bp and 70 bp).

**Figure 2:**
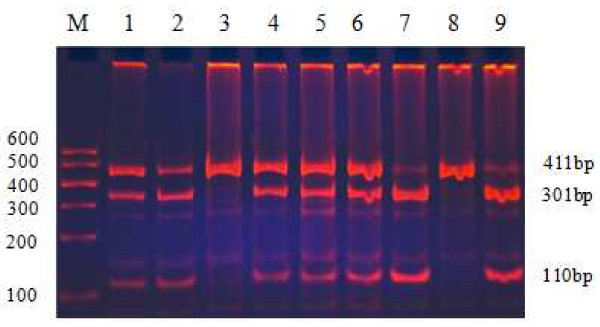
**Genotyping of PCR products of LIPC-250 G/A.** Lane M, 100 bp Marker Ladder; Lanes 1/2/4/5/6, GA genotype (411 bp, 301 bp and 110 bp); Lane 3/8, GGgenotype (411 bp); and Lanes 7/9, AA genotype (301 bp and 110 bp).

**Figure 3:**
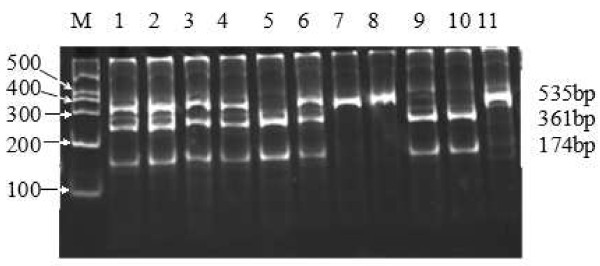
**Genotyping of PCR products of CETPTaq1B.** Lane M, 100 bp Marker Ladder; Lanes 1-4/6, B1B2 genotype (535 bp, 361 bp and 174 bp); Lane 7/8/11, B1B1genotype (535 bp); and Lanes 5/9/10, B2B2 genotype (361 bp and 174 bp).

The remaining 2 ml blood sample was used to extract genomic DNA by phenol-chloroform methods. The isolated DNA was stored at 4°C until analysis. All genotypes had been examined using restriction fragment length polymorphism (RFLP) amplified by polymerase chain reaction (PCR). Information on polymorphic sites of ABCA1, LIPC and CETP was from the database of single nucleotide polymorphisms (SNPs) established by the National Center for Biotechnology Information. The ABCA1 R219K polymorphism was investigated by using the following primers: 5'-GTA TTT TTG CAA GGC TAC CAG TTA CAT TTG ACAA-3'(forward) and 5'-GAT TGG CTT CAG GAT GTCC-3'(reverse), according to previous literure [65]. The detection of genotypes was performed using 8% polyacrylamide gel electrophoresis. The SNP rs2230806 predicts a non-synonymous G to A exchange in exon 7 of ABCA1 gene resulting in a R (Arg) to K (Lys) amino acid exchange at position219. After digestion with EcoNI, three different fragments: 177 bp or 107 ,70 bp were generated. For the LIPC-250 G/A and CETP Taq1B polymorphisms, PCR amplification was performed using 5'-GGCAAGGGCATCTTTGCTTC-3'(forward)/ 5'-GGTCGATTTACAGAAGTGCTTC-3'(reverse) [66] and 5'-CACTAGCCCAGAGAGAGGAGTGCC-3'(forward)/5'-CTGAGCCCAGCCGCACACTAA-3'(reverse) [67], respectively. The LIPC-250 G/A products were 411, 301 and 110 bp or 301 and 110 bp or 411 bp by restriction enzyme DraI. The PCR amplification of CETP Taq1B was catalyzed by TaqI to generate 535 bp, 361 bp and 174 bp or 361 bp and 174 bp or 535 bp (Figures 1, 2, 3).

### Statistical analyses

Quantitative variables were expressed as mean±standard deviation, and qualitative variables as percentages. First, quantitative data was performed for normality test, if not, transformed into natural logarithm profile. The comparison of quantitative traits, including biochemical index and cognitive function scores (MMSE, WMS and WCST) between AD group and controls was carried out using Independent-Samples *t*-Test. The effects of three SNPs on serum lipid levels, the age-at-onset and cognitive function scores were investigated by one-way ANOVA. Univariate analysis of variance was applied to assess the interactive effect of these gene variants on lipid profiles. Chi-square(χ^2^) test was used to compare genotype and allele frequency differences between AD cases and controls. Standard goodness-of-fit test was used to test the Hardy-Weinberg equilibrium. Multivariate logistic regression analysis was done in order to evaluate the simultaneous influence of these genetic variations and some environmental factors on the risk of AD. The estimated odds ratios (ORs) were adjusted for the effect of significant covariates, such as age, gender and education. All statistical tests were two-sided. A P value of less than 0.05 was considered statistically significant. All statistical tests were carried out using SPSS 13.0.

## Results

### General characteristics and serum lipid levels

There were no significant differences in age, gender and education structure, systolic blood pressure, fasting blood glucose, TG, LDL-C, apoA-I, apoB levels and the percentages of history of smoking, alcohol, diabetes, hypertension and CAD between the two groups in the present study (*P* > 0.05). The levels of diastolic blood pressure and TC, the percentages of history of hyperlipidemia in AD group were higher than that in controls, whereas HDL-C level was lower than that of controls (*P <* 0.05) (Table 1).

**Table 1:** Comparison of general characteristics and serum lipids between AD patients and controls

**Variable**	**Cases**	**Controls**	***t*-value or χ**^**2**^	***P***
N	104	104		
Age(years)	77.8±6.74	76.5±6.14	−1.495	0.137
Male/female	57/47	56/48	0.019	0.889
Systolic blood pressure(mmHg)	138.1±19.5	134.7±19.5	−1.239	0.217
Diastolic blood pressure(mmHg)	79.6±9.56	76.6±9.65	−2.256	0.025
Cigarette smoking[n(%)]	27(26.0)	40(38.8)	3.918	0.048
Alcohol consumption[n(%)]	8(7.7)	6(5.8)	0.286	0.593
History of hypertension[n(%)]	58(56.3)	64(64.6)	1.466	0.226
History of hyperlipidemia[n(%)]	44(42.3)	27(26.0)	6.180	0.013
History of diabetes[n(%)]	26(25.0)	15(15.2)	3.052	0.081
History of CHD[n(%)]	46(44.7)	41(41.4)	0.217	0.641
Education(proportion of high school and above level)	17(16.3)	36(34.6)	9.140	0.003
TG(mmol/L)	1.36±0.62	1.32±0.71	−0.909	0.364
TC(mmol/L)	4.48±0.89	4.10±1.04	−2.826	0.005
HDL-C(mmol/L)	1.33±0.31	1.44±0.31	2.450	0.015
LDL-C(mmol/L)	2.46±0.66	2.40±0.82	−0.848	0.397
ApoA-I(g/L)	1.17±0.28	1.21±0.22	1.162	0.247
ApoB(g/L)	0.79±0.22	0.80±0.22	0.089	0.929

TC, Total cholesterol; TG, Triglycerides; HDL-C, highdensity lipoprotein cholesterol; LDL-C, low-density lipoprotein cholesterol; Apo, Apolipoproteins.

### Comparison of genotypes and alleles between cases and controls

Three ABCA1R219K genotypes(RR, RK, KK), three LIPC-250 G/A genotypes (GG, GA, AA) and CETPTaq1B genotypes (B1B1, B1B2, B2B2) were detected and their genotype distributions in cases were consistent with Hardy–Weinberg equilibrium (R219K, χ^2^ = 1.674, df = 2, *P* = 0.433; LIPC-250 G/A, χ^2^ = 4.163, df = 2, *P* = 0.125; CETPTaq1B, χ^2^ = 2.047, df = 2, *P* = 0.359), but in controls, with the exception of ABCA1R219K (χ^2^ = 0.539, df = 2, *P* = 0.764), the polymorphisms of -250 G/A and Taq1B both departed from HWE.

ABCA1R219K: There was significant difference in the frequencies of three genotypes between the AD patients and controls (χ^2^ = 8.777, *P* = 0.012). The frequency of KK genotype in AD group was obviously lower than that in controls (χ^2^ = 5.261, *P* = 0.022). The frequency of K allele (KK + RK genotype) in AD patients was 54.8%, significantly lower than that of controls (70.2%, *P* = 0.022). No significant difference in R or K allele frequency was found between the two groups (*P* > 0.05).

LIPC-250 G/A: Significant differences in the frequencies of three genotypes (GG,GA and AA) between the AD patients and controls were determined (χ^2^ = 7.925, *P* = 0.019). The frequency of GG genotype in AD group was obviously higher than that of controls (27.9%&13.5%, χ^2^ = 5.233, *P* = 0.022). The frequency of GA + AA genotype in AD patients was significantly lower than that of controls (72.1%&86.5%, *P* = 0.016). There was no significant difference in the frequency comparison of G allele and A allele.

CETPTaq1B: The genotype distributions of the Taq1B polymorphism in AD cases differed from that of controls (χ^2^ = 8.102,*P* = 0.017). AD cases showed significantly higher B1B1 genotype frequency compared to controls (27.9% versus 14.4%; *P* = 0.035). No significant difference between both groups was determined in allele frequencies (*P* > 0.05). Distribution of all genotypes and alleles were showed in Table 2.

**Table 2:** Genotype distribution and allele frequencies of these three polymorphisms (ABCA1R219K, LIPC-250 G/A and CETP Taq1B) in cases and controls

**Genotypes and alleles**	**AD patients (n/%)**	**Controls (n/%)**	**χ**^**2**^	***P***
ABCA1R219K				
RR	47(45.2)	31(29.8)	3.282	0.070
RK	51(49.0)	56(53.8)	0.234	0.629
KK	6(5.8)	17(16.3)	5.261	0.022
	χ^2^ = 8.777 *P* = 0.012			
R/K(%)	70/30	57/43	3.646	0.056
LIPC-250 G/A				
GG	29(27.9)	14(13.5)	5.233	0.022
GA	64(61.5)	82(78.8)	2.219	0.136
AA	11(10.6)	8(7.7)	0.474	0.491
	χ^2^ = 7.925 *P* = 0.019			
G/A(%)	59/41	53/47	0.731	0.393
CETP Taq1B				
B1B1	14(13.5)	9(8.7)	1.087	0.297
B1B2	61(58.7)	80(76.9)	2.560	0.110
B2B2	29(27.9)	15(14.4)	4.455	0.035
	χ^2^ = 8.102 *P* = 0.017			
B1/B2(%)	57/43	53/47	0.323	0.570

### SNPs and serum lipid levels

For the first time we performed genetic analysis of several common polymorphisms of ABCA1, LIPC and CETP in AD patients and control subjects for the association with serum lipid levels, and elucidated that the important genetic factors could influence cholesterol levels in our population. For ABCA1 R219K polymorphism, there were significantly higher levels of HDL-C and apoA-I in the carriers of KK genotype and K allele (*P* < 0.05). During RR,RK,KK genotypes, the levels of HDL-C and apoA-I significantly increased in ascending order (*P* < 0.05), with TG level decreased but no statistically significance found (*P* > 0.05). We didn't found any positive association between R219K SNP and plasma levels of TC,LDL-C and apoB. In all serum lipids, only apoA-I had been found distinct relevance with LIPC-250 G/A. The level of apoA-I was highest in AA genotype, followed by GA and GG genotypes in total subjects. The subjects carrying A allele had higher plasma apoA-I level than non-carriers in both whole population and the control group. Nevertheless, no significant association was reached in our study for serum HDL-C level with SNP of -250 G/A. In addition, B2B2 genotype of CETP Taq1B showed significant association with higher plasma HDL-C levels than other genotypes (*F* = 5.598, *P* = 0.004). After stratifying by gender, this change was only found in females (*F* = 3.404, df = 2, *P* = 0.038). And there was obviously lower HDL-C levels in B1 allele carriers (1.24±0.35 mmol/L) than in non-carriers (1.45±0.35 mmol/L, *t* = −2.463, *P* = 0.015) (Table 3).

**Table 3:** SNPs and serum lipid levels and age-on-set

**Parameter**		**ABCA1R219K**				**LIPC-250 G/A**				**CETP Taq1B**			
		**RR**	**RK**	**KK**	**RK + KK**	**GG**	**GA**	**AA**	**GA + AA**	**B1B1**	**B1B2**	**B2B2**	**B1B2 + B1B1**
Serum lipid levels	TG (mmol/L)	1.39 ± 0.80	1.33 ± 0.57	1.20 ± 0.60	1.31 ± 0.58	1.24 ± 0.63	1.35 ± 0.69	1.50 ± 0.55	1.37 ± 0.68	1.25 ± 0.53	1.38 ± 0.70	1.32 ± 0.71	1.34 ± 0.67
	TC (mmol/L)	4.22 ± 0.97	4.34 ± 0.99	4.31 ± 0.96	4.34 ± 0.99	4.12 ± 0.89	4.32 ± 0.99	4.45 ± 1.03	4.34 ± 1.00	4.21 ± 0.98	4.30 ± 0.95	4.39 ± 1.18	4.28 ± 0.96
	HDL-C (mmol/L)	1.27 ± 0.28	1.39 ± 0.24^a^	1.76 ± 0.41	1.45 ± 0.31^a*^	1.35 ± 0.33	1.39 ± 0.30	1.41 ± 0.37	1.40 ± 0.31	1.37 ± 0.43^c^	1.21 ± 0.32	1.46 ± 0.35^c*^	1.24 ± 0.35^d^
	LDL-C (mmol/L)	2.35 ± 0.72	2.49 ± 0.76	2.44 ± 0.75	2.48 ± 0.75	2.29 ± 0.73	2.45 ± 0.74	2.58 ± 0.75	2.47 ± 0.74	2.38 ± 0.73	2.45 ± 0.73	2.44 ± 0.86	2.43 ± 0.73
	apoA-I (g/L)	1.11 ± 0.25	1.20 ± 0.23^a^	1.39 ± 0.25	1.24 ± 0.25^a*^	1.11 ± 0.25	1.20 ± 0.24^b^	1.29 ± 0.34^b*^	1.21 ± 0.25^b^	1.22 ± 0.29	1.17 ± 0.24	1.25 ± 0.28	1.18 ± 0.25
	apoB (g/L)	0.79 ± 0.23	0.80 ± 0.22	0.78 ± 0.23	0.80 ± 0.22	0.77 ± 0.25	0.80 ± 0.22	0.83 ± 0.22	0.80 ± 0.22	0.78 ± 0.20	0.79 ± 0.23	0.84 ± 0.26	0.79 ± 0.22
Age-on-set (years)		73.2 ± 7.89	72.6 ± 6.92	73.2 ± 6.43	72.6 ± 6.82	73.2 ± 8.36	73.1 ± 6.87	70.9 ± 7.34	72.8 ± 6.94	73.0 ± 7.04	72.8 ± 7.76	72.9 ± 6.04	72.9 ± 7.49

^a^*P* < 0.05 in comparison with RR genotype, ^a*^P < 0.01 in comparison with RR genotype.

^b^*P* < 0.05 in comparison with GG genotype, ^b*^P < 0.01 in comparison with GG genotype.

^c^*P* < 0.05 in comparison with B1B2 genotype, ^c*^P < 0.01 in comparison with B1B2 genotype.

^d^*P* < 0.05 in comparison with B2B2 genotype.

We have further assessed the impact of the combined effects of these frequent single-nucleotide polymorphisms (SNPs) in the HDL catabolic pathway on the lipid levels. There existed evident combined genotype effect of the ABCA1 R219K and the LIPC-250 G/A polymorphisms on HDL-C levels in total subjects: the carriers of the KK/AA genotype showed the highest levels of HDL-C (2.07 +/− 0.11 mmol/L), whereas those carrying the RR/GG genotype showed the lowest (1.07+/− 0.22 mmol/L) (Table 4). There was no significant difference of HDL-C level among subjects carrying CETP Taq1B genotypes with others.

**Table 4:** Combination of ABCA1 R219K and LIPC -250 G/A variants and serum HDL-C levels(mmol/L)

		**LIPC-250 G/A genotypes**		
		**GG**	**GA**	**AA**
ABCA1 R219 K genotypes	RR	1.07±0.22	1.33±0.82	1.19±0.26
	RK	1.40±0.26	1.36±0.24	1.52±0.19
	KK	2.02±0.66	1.70±0.40	2.07±0.11
		*F =* 3.930 *P* = 0.004		

### SNPs and age-on-set and cognitive test scores

We examined the association of these polymorphisms with age-on-set of AD cases, but found no significant result. We also determined the association of these genetic variations with quantitative measures of cognition such as MMSE, WMS and WCST scores. In whole population, subjects carrying ABCA1 219 K allele or LIPC -250A allele obtained higher MMSE or WMS scores than non-carriers, however, no significant association was observed in AD group or controls. We didn't find any effect of CETPTaq1B polymorphism on cognitive test scores.

### Logistic regression analysis:risk factors for AD;simultaneous effects of ABCA1,LIPC and CETP

Unconditional logistic regression analysis was done for the assessment of the influence of age, gender, education, TC, HDL-C and ABCA1, LIPC, CETP minor alleles, and the interaction terms (ABCA1 × LIPC × CETP) on the risk for developing AD. The analyses revealed no significant influences of CETPTaq1B polymorphism, TC and HDL-C on the risk of AD. It was evident that, while there was no obvious interaction, both minor alleles (ABCA1 and LIPC) had statistically significant independent effects on AD risk (*P* = 0.005, OR = 0.405, 95%CI:0.217-0.758 and *P* = 0.018, OR = 0.405, 95%CI:0.191-0.858, respectively). Moreover, it was also suggested that age (*P* = 0.003, OR = 2.620, 95%CI:1.381-4.972) and levels of education(B = −1.052, adjustedOR = 0.349, P = 0.004, 95%CI:0.172-0.710) had an independent significant association with AD respectively. The final model is shown in Table 5.

**Table 5:** Logistic regression analysis:risk factors for AD

**Variable**	**B**	***P***	**OR**	**95%CI**
age	0.963	0.003	2.620	1.381-4.972
education	−1.052	0.004	0.349	0.172-0.710
219K allele	−0.903	0.005	0.405	0.217-0.758
−250A allele	−0.905	0.018	0.405	0.191-0.858

## Discussion

Alzheimer's disease is a multifactorial disease but its aetiology and pathophisiology still has not been fully understood. Similar to most complex diseases, AD is likely to be influenced not only by genetic factors, but also by environmental components. One environmental factor which role in AD pathogenesis remains controversial based on systematic review of case–control and cohort studies is dyshomeostasis of cholesterol [9]. It is well known that dyslipidaemia is a complex trait caused by multiple environmental and genetic factors and their interactions. This study aimed to investigate variations of several cholesterol-related genes and correlation to serum lipid levels and risk of LOAD in Chinese Southern Han population in Hunan Province.

The relationship between cholesterol levels in plasma and risk of AD has been still a matter of discussion. In our study, the level of TC in AD patients was higher than that in controls (*P <* 0.01), whereas HDL-C level lower than that in controls (*P <* 0.05), in line with previous studies indicating lower plasma HDL-C levels, higher LDL-C levels, or increased TC level in AD cases when compared to controls [11,68,69]. But after adjusting other factors in logistic regression analysis we didn't find any potential association of serum TC or HDL-C with risk of AD. Hall K et al. [12] and Helzner EP [4] et al. respectively had reported an obvious association of plasma TC or LDL-C levels with increased risk of LOAD and the rate of cognitive decline in the eldly, but the former result only for carriers without APOEε4 [12]; Recently higher HDL-C level (>55 mg/dL) was demonstrated with lower risk for AD in a large prospective cohort study carried out in 1130 cognitively intact old people by Reitz C et al. [10]. However, this was contradicted by some studies demonstrating increased cholesterol level was linked to reduced AD risk [70,71]. Furthermore, high midlife TC, not late-life TC, was associated with increased risk of AD and cognitive decline, that was supported by a systematic review of prospective studies with meta-analysis [14]. It remained controversial due to ethnic or region specificity, carrying apoEε4 allele or not, difference of life styles such as fat intake and BMI [72,73], physical activity and oxidative stress, as well as homocysteine by likely impairing apoE3 function for HDL [74]. It was reported that using some drugs might produce any uncertain effects, for example, taking statins could slow the rate of cognitive decline and delay the onset of AD and reduce risk of dementia in the eldly [17,75], and donepezil hydrochloride using for AD patients may cause up-alteration of serum TG,TC and LDL-C [76]. These factors may be of equal importance when considering AD risk. Although numerous hypotheses have been advanced to explain the role of cholesterol in the pathogenesis, it remained to be elucidated. Currently, it has been extensively recognized that cholesterol may be associated closely with brain Aβ levels. Animal studies showed that hypercholesterolemia may lead to increase brain Aβ levels [20]. Recent convincing evidence by Lesser et al. performed in 281 AD patients indicated that serum TC and LDL levels were clearly positively correlated with densities of neuritic plaques in both neocortex and hippocampal regions [77]. Further research to determine if dyslipemia results in a decreased risk of LOAD would strengthen these findings.

In this pilot study we determined an apparent association between the polymorphism of ABCA1 R219K and AD. The frequencies of carrying both KK genotype and K allele in AD group were obviously lower than that of controls (χ^2^ = 5.261, *P* = 0.022), and logistic regression analysis showed that K allele may be an independent protective factor for AD (*P* = 0.005, OR = 0.405, 95%CI:0.217-0.758), consistent with previous report by Wang F et al. that risk of AD was significantly decreased in K allele or KK homozygote carriers compared with RR genotypes carriers [57]. Nevertheless, some studies in other populations have excluded the association of ABCA1 gene with LOAD [38,78], while others have shown an increased risk of AD for female 219 K allele carriers [56]. Important differences in the population sizes, genetic composition, and background environment of the populations studied such as diets may be partly responsible for mutative AD susceptibility and these discordant results. The minor allele frequency of R219K was reported 25% in Caucasian populations [79], 46% in Europeans [65]. In addition, we didn't find any correlation with age-on-set of AD, however, Wollmer MA et al. once reported that A allele was associated with delayed age-at-onset by 1.7 years on average [38].

Mechanisms via which this polymorphism could affect pathogenesis of AD remain to be elucidated. Some studies focused on pathological mechanism have suggested that ABCA1 mRNA expression was significantly elevated at the earliest stage of dementia, and positively correlated with neuropathological stages and neuritic plaque density counts and dementia severity, through comparison of postmortem hippocampus from persons at different stages of AD and cognitively intact normal donors by Akram A et al. [31]. And transgenic animal models have repeatedly shown the influence of loss of ABCA1 on apoE lipidation, Aβ production or deposition and cognitive impairment [80]. Secondly, ABCA1 gene is located on 9q31.1, just closing to susceptible loci region of LOAD oncome [81]. Furthermore, K allele may produce a benefit profile of serum lipids [82]. Therefore, it is also understandable that carrying K allele may be correlated with protecting from AD. However, much larger sample would be needed to confirm such an interaction.

ABCA1 is a member of a superfamily of membrane proteins as a key enzyme in regulating plasma HDL-C and apoA-I metabolism [83]. Many conflicting results from previous studies have evaluated the associations of R219K polymorphism with level of HDL-C and risk of developing CAD [82,84,85]. However, little work about the association of this genetic variation with plasma lipids in AD patients has been assessed. In this pilot study, we first reported the association of R219K gene variants with lipids in AD patients, suggesting the possible involvement of cholesterol with AD. There was significantly higher levels of HDL-C and apoA-I in the carriers of KK genotype and K allele (*P* < 0.05) in either AD patients or controls, similar to several previous findings [82,86,87]. Recent meta-analysis using thousands of samples established that this association only presented in Asian, not in White populations [82,86]. Genvigir FD et al. determined that K allele was correlated with higher apoA-I level in Brazilian individuals [88]. However, other studies failed to find any significant influence of R219K variation on HDL-C or other lipid parameters levels [85,89-91]. The explanation for these contradictory findings has been set on not only the limited effects of gene variation but gene-environment interactions. Different populations have discrepant HDL-C levels, such as relatively lower in Turks [92]. KK genotype of ABCA1R219K polymorphisms has been reported linked to a 5.5% higher concentration of small HDL particles [93]. Moreover, controversial conclusion may vary with other factors such as age [94], gender [95], smoking [96], obesity [97]and existing linkage disequilibrium or interactive effect with other candidate genes [98]. In addition, the influence of ABCA1 gene variants on other lipid molecules and enzymes secondarily can mildly influence HDL concentration. Clee SM, Pasdar A and Li J et al. reported downward trend of TG level during RR,RK,KK genotypes and 219 K allele carriers [65,99,100]. This trend was replicated in our population, but no statistically significance found. Further investigations are needed to prove the influence of ABCA1 polymorphisms on serum lipid levels and to determine whether it could be a genetic determinant of AD.

Previous studies have shown that the A allele of the LIPC-250 G/A and the B1 allele of Taq1B in the intron region of CETP were associated with decrease in HL activity and CETP activity and elevation of HDL-C levels [49,101]. The present study first revealed the association of -250 G/A variants with plasma lipids in AD patients in the Chinese population, only finding an significant effect on serum apoA-I levels, which was higher in the subjects carrying A allele or AA genotype than other genotypes or alleles carriers in total subjects and the control group. It was similar to the previous research studied in type2 diabetes and CAD patients by Wei M et al. [52]. Nevertheless, we found no significant effect on HDL-C and other lipoprotein levels. To date, there has been considerable evidence about -250 G/A variant and plasma lipoprotein concentrations, but the result remains inconsistent [52,102-104]. Influence of gender-specificity on the association was found in some studies [51,103,104]. Transgenic mice studies has demonstrated that overexpression of HL has led to a marked reduction in plasma HDL-C [105], however, there have existed many contradictory results in clinical epidemiological studies, which may be attributed to various factors, such as (i)restriction in terms of sample size and ethnic diversity; (ii)the integrated effects of multiple polymorphisms of genes, for instance, Wood KC et al. indicated interactions during LIPC-514C/T and LIPC-250 G/A and apoE gene polymorphisms could distinctly influence serum lipid profiles [106]; (iii)both having pro-atherogenic and anti-atherogenetic properties for HL [107,108]; (iv)related to HDL subclasses, mainly as HDL_2_-C [49], etc. The proportion of variation in HDL-C attributable to polymorphisms within the HL gene is probably no more than 25% [40,109]. However, it is not clear whether the promoter polymorphisms was functional or merely a marker of another physiological polymorphism located elsewhere, because of complete linkage disequilibrium [47].

CETP has its capacity to mediate transferring of neutral lipids among the lipoproteins by reversely exchanging triglycerides and cholesteryl ester between TG-rich lipoproteins and HDL, producing HDL with reduced cholesteryl ester and increased TG [110]. Most previous studies examining the relationship between CETP variants and plasma HDL-C levels and CAD risk have focused on the intronic Taq1B variant [101]. Our research first indicated that B2B2 genotype was significantly associated with higher plasma HDL-C levels than other genotypes, and HDL-C levels in B1 allele carriers (1.24±0.35 mmol/L) was obviously lower than that in non-carriers (1.45±0.35 mmol/L, *P* = 0.015) in AD patients, supporting previous and recent studies in which homozygous minor allele genotypes or B2 allele would be associated with highest HDL-C concentrations [43,44,98,111]. However, there also have been addressed no distinct anti-atherogenic potential of this polymorphism in some studies [102,112]. It may be due to diverse genotype or allele frequencies in different racial and regional groups. The meta-analyse in 2008 suggested the minor allele (B2 allele) frequency in the Whites was the same as East Asian populations (0.42) [101], in accordance with 0.43 and 0.47 respectively in our AD participants and controls. We also found the effect of B2 allele or homozygous genotype on HDL-C more apparent in females, which was probably associated with modification of hormone in menopausal status [113]. Such genetic testing is limited by the fact that each sequence variant explains only a modest fraction of the variance (2% or less) in lipid levels [114]. However, the combination of several lipid-related polymorphisms perhaps could improve risk prediction.

CETP-mediated triglyceride enrichment of HDL-C notably increases the ability of hepatic lipase to assemble HDL-C [41]. So the potential interaction between LIPC and CETP variants on lipid levels needed to be elucidated. But our study indicated that there was no significant effect of combinations of CETPTaqIB and LIPC -250 G/A or ABCA1 R219Kvariants on plasma HDL-C levels and other lipids. We found a significant interaction of ABCA1 R219K and LIPC-250 G/A polymorphisms on HDL-C levels in the total subjects (*F* = 3.930, *P* = 0.004). Previous studies have shown that combinations of minor variants in these common polymorphisms could produce more pronounced effect on serum lipids [43,98,115]. However, recently a large cardiovascular cohort by Kathiresan et al. suggested that combinations of common SNPs only showed a weak association with alteration in HDL-C level [116]. The determinants of HDL-C levels are involved in not only gene variants, but also many environmental profiles. Approximately 50% of the inter-individual variation in serum HDL-C levels was considered to be genetically determined [117]. In addition, age, diet, smoking, alcohol, body mass index (BMI), physical activity [118] and different levels and distribution of HDL subclasses all play a role in determining individual HDL-C levels. CETP and HL are implicated in the metabolism of plasma lipoproteins, but the effects of CETP and LIPC genotypes on atherosclerosis may be dependent on LDL-receptor activity [119]. The relationship between HDL and these gene variants needs to be validated in a larger longitudinal study.

In the past decade, while a number of case–control studies have been carried out to investigate the relationships between LIPC or CETP polymorphisms and risk of atherosclerosis diseases [43,51,52,101,115], little is known about their effect on neurodegenerative disease. In 2008 Haiyan Zhu et al. has detected three LIPC SNPs(rs6083,rs6084 and rs6074 in coding region) in three LOAD series, and recently in a German case–control sample (438 AD patients and 290 controls) Laws SM et al. also evaluated 25 single nucleotide polymorphisms(−250 G/A not included), however, no association with AD was found [60,61]. Our study first showed that -250 G/A variant may decrease the risk of AD after adjusting other factors like gender, age and education for logistic regression analysis. The independent protective effect of A allele may be understandable due to its implication in lipid metabolism involved in decreasing plasma HL activity, elevating HDL-C and apoA-I levels. There was significant difference in the A allele frequency in different regions and populations: 0.21 in American Caucasian, 0.47 and 0.45 in Japanese Americans and African Americans respectively [42,49], 0.63 in Korean [42] and 0.32 in Southern Brazilian population [51]. Moreover, the influences of APOEε4 and other genes cholesterol-related need to be taked into account.

Earlier research showed that in AD tissue CETP was extensively expressed in the astrocytes of gray matter as well as the white matter [120], so alteration of CETP activity might play a role for AD pathology. Several studies have showed CETP gene variants such as I405V,D442G may have a potential association with memory decline and dementia risk [59,121,122]. But others didn't find any positive results [123,124]. For TaqIB polymorphism, subsequent studies reported no statistically significant differences with respect to either genotype or allele frequencies between CETP TaqIB and AD [125]. And the same results maintained in the study of Xuanwu Hospital of our country by Chen DW et al. regardless of APOE ε4 carrier status [59]. We also didn't find any correlation between TaqIB variation and AD risk in Hunan Han population, though AD cases showed significantly higher B1B1 genotype frequency compared to controls (27.9% versus 14.4%; *P* = 0.035). Further studies combined with multi-ethnic research are warranted to determine the value of CETP variants for AD.

We further investigated interactive effect among these ABCA1 and LIPC and CETP variants on the risk of AD. These variants, which could decrease protein activity and increase HDL-C levels, should, in theory, decrease atherosclerosis and reduce the incidence of AD. However, in our case–control study, on adjusting for age, sex and education, logistic regression analysis revealed no statistically significant interaction.

In this pilot study, we first determined the association of these genetic variations with quantitative measures of cognition such as MMSE, WMS and WCST scores. The former two ones were used to examine the degree of memory impairment, and WCST implicated in evaluation of frontal behavioral function. ABCA1219K allele and LIPC-250A allele carriers were associated with higher MMSE or WMS scores in total sample, however, no significant difference was observed in AD patients or controls. This was in line with indication in logistic regression. This protective effect may be attributed to the beneficial effect of the ABCA1219K and LIPC-250A alleles on HDL-C. Pathological mechanism studies suggested ABCA1mRNA expression was positively correlated with Braak neuropathological stages and dementia severity [31]. And Hirsch-Reinshagen et al. has found that increased ABCA1 expression may result in improved cognition in amyloid mouse models [80]. However, there was no significant association of ABCA1 gene variants with MMSE scores in the literature [56]. Replication with a larger sample size and in other ethnic groups and standardization of test process is warranted.

Many environmental vascular risk factors such as age, smoking, obesity, abnormal lipid metabolism, physical inactivity and diabetes are thought to be necessary co-contributors or initiators of the disease process. We didn't find smoking, alcohol and diabetes had any distinct effect on AD incidence as well as sex. However, some studies have suggested female gender was associated with an increased risk of AD [78,126]. Age did not significantly influence the risk of AD in the study consisted of 241 German AD patients and 294 non-demented controls by Kölsch H et al. [78].

Currently, while the effect of education on the incidence of dementia and AD has not been stated universally, major studies reported to date as well as our finding have showed that higher education might shorten cognitive impairment and decrease AD risk. We found higher education may be an independent protective determinant of AD, in accordance with the results in previous studies and the recent Epidemiological Clinicopathological Studies in Europe (EClipSE) that longer years in education were associated with decreased dementia risk and low educational attainment was associated with a higher risk of AD [126-128]. Reuser M et al. also found that higher education may delay incidence of cognitive decline and protect against cognitive impairment between age 55 and 75 by assessment in three ethnic groups in the US Health and Retirement Study [129]. But adjusting for age the Framingham Study by Cobb JLet al showed low educational attainment was not a risk factor for AD compared with those who earned a high school diploma(relative risk:1.04, 95% CI:0.62 to 1.74) [130]. Further investigations are needed to prove the influence of education on risk of AD in Chinese Han population.

## Conclusion

In summary, for the first time we reported a significant association of the ABCA1 R219K and LIPC-250A polymorphisms with sporadic AD risk and cognitive and memorial scores in Southern Chinese Han population, but no pronounced effect in CETP TaqIB SNP. We also provided preliminary evidence for the first time that these three variations were associated with serum lipids in AD patients. Hence, the combined study of common genetic variants as well as environmental risk factors, may contribute to the etiological understanding of the remaining fraction unexplained in AD. Replication with a larger sample size, more detailed individual information and more rare loci variants as well as other ethnic groups is warranted.

## Competing interests

The authors declare that they have no competing interests.

## Authors’ contributions

JW, PW and ZX contributed to the design of the study. JW and PW contributed to the collection of all samples. JW, PW and ZX contributed to the analysis and interpretation of data. WC, WC and HZ contributed to the financial support. JW was responsible for finishing the article including all figures and tables. All authors contributed to critical revision of the paper. All authors have read the manuscript and gave their final approval for this version of the manuscript to be published.
